# P30 A case of small bowel and leukocytoclastic vasculitis associated with acitretin therapy

**DOI:** 10.1093/rap/rkac067.030

**Published:** 2022-09-28

**Authors:** Helen Wanstall, Mithun Chakravorty, Mohummad Shaan Goonoo, Ian Bickle, Ruth Macinerney, Kevin Fairburn, Karolina Nemeth-Roszpopa

**Affiliations:** Sheffield Teaching Hospitals, Sheffield, United Kingdom; Royal Derby Hospital, Derby, United Kingdom; Sheffield Teaching Hospitals, Sheffield, United Kingdom; Rotherham Foundation Trust, Rotherham, United Kingdom; Chesterfield Royal Hospital, Chesterfield, United Kingdom; Chesterfield Royal Hospital, Chesterfield, United Kingdom; Chesterfield Royal Hospital, Chesterfield, United Kingdom

## Abstract

**Introduction/Background:**

Acitretin is an oral retinoid that is used to treat a number of skin conditions including acne. It is generally well tolerated and common side-effects such as gastrointestinal upset, mucosal and skin reactions are usually mild. However, we present what we believe to be the first reported case of small bowel and leukocytoclastic vasculitis (LCV) caused by acitretin and this required prompt treatment with intravenous glucocorticoids and cyclophosphamide to induce remission.

**Description/Method:**

A 53-year-old caucasian male presented to the Emergency Department with severe bilateral foot pain for four days and a purpuric rash which initially affected the soles of his feet and spread to affect all four limbs, both hands and abdomen. He was three weeks into his second course of acitretin 20mg daily for a flare of psoriasis, having ceased treatment three months before due to a suspected iatrogenic rosacea type reaction. He had generalised abdominal pain and recent constipation. Observations demonstrated a sinus tachycardia at 130 beats per minute only and examination revealed widespread patchy non-blanching palpable purpura and deep ulcers over the soles of both feet. There was mild abdominal distension and global tenderness but no peritonism. The remainder of the physical examination was unremarkable. Initial blood tests showed: haemoglobin 156 g/L, white cell count 13 x 10^9^/L (neutrophilia: 10.6 x 10^9^/L), platelets 514 x 10^9^/L, CRP 123 mg/L, ESR 12 mm/hr, normal renal and liver function. Detailed immunology, microbiology and viral screens were negative including anti-neutrophil cytoplasmic antibodies (ANCA) and cryoglobulins. Urine dipstick was unremarkable. Abdominal x-ray showed distended small bowel loops and CT demonstrated significant intestinal wall oedema suggestive of small bowel vasculitis. Skin biopsy demonstrated typical changes of leukocytoclastic vasculitis. After surgical review he was managed conservatively on the high dependency unit with intravenous methylprednisolone 1g daily for three days followed by intravenous hydrocortisone and one dose of cyclophosphamide (15mg/kg). Total parenteral nutrition was initiated, and his bowel symptoms settled after two weeks. His skin significantly improved and he was discharged after four weeks’ inpatient stay. Actretin was stopped indefinitely, and he has remained in remission over three years later. Prednisolone was successfully tapered at a slow rate, and no further cyclophosphamide was required. His psoriasis is currently managed well with oral methotrexate.

**Discussion/Results:**

LCV is a small vessel vasculitis which mostly occurs in the cutaneous form but can be life-threatening with systemic involvement. Around 50% of cases are idiopathic, with infections (15-20%), autoimmune diseases (15-20%) and malignancy (<5%) being well-recognised causes.

Drug-induced LCV accounts for nearly 15% of cases and antibiotics, non-steroidal anti-inflammatories, anti-epileptics and TNF-alpha blockers are well-known precipitants. It usually occurs within three weeks after commencement of the medication, and the timing of re-introduction of acitretin in our patient makes it likely to be the cause. Up to 90% of LCV cases resolve within weeks to months after addressing the precipitating cause, with or without oral steroids. However, a 2% mortality is reported, likely due to systemic involvement and given the severity of our patient’s presentation, it was decided to commence immunosuppression in view of the skin biopsy and CT findings. (A biopsy of the small bowel was considered but would have delayed treatment).

To our knowledge there are no published case reports linking LCV and small bowel vasculitis to acitretin therapy. However, there have been several case reports of LCV and other vasculitides caused by all-trans-retinoic acid (ATRA) and isotretinoin (first generation retinoids). Yamada et al. (1999) reported a severe case of haemonecrosis of the ileum, as a complication of ATRA treatment for acute promyelocytic leukaemia. Surgical intervention was required and histology demonstrated LCV.

Isotretinoin was attributed to cause 5 out of 11 LCV cases reviewed by Dwyer et al. (1989). Turan et al. (2012) reported a case of a Henoch-Schonlein Purpura secondary to isotretinoin that improved with discontinuing treatment and three weeks of fluocortolone 20mg daily. More recently, a case of diffuse alveolar haemorrhage due to isotretinoin-induced granulomatosis with polyangiitis was described by Assuncao et al. (2018), which remitted with intravenous methylprednisolone and cyclophosphamide.

**Key learning points/Conclusion:**

Although very rare, consider retinoids including acitretin in cases of drug-induced LCV and small bowel vasculitis.

Symptoms usually improve on cessation of the treatment alone, but immunosuppression might be required for more severe cases.

Future case series could help guide treatment choices.

